# Toll-like receptor 4 in phagocytosis of *Escherichia coli *by endotoxin-activated human neutrophils in whole blood

**DOI:** 10.1186/cc11767

**Published:** 2012-11-14

**Authors:** I Prokhorenko, S Zubova, D Kabanov, E Voloshina, S Grachev

**Affiliations:** 1 Institute of Basic Biological Problems, Pushchino, Moscow Region, Russia

## Background

Endotoxic shock during infection by Gram-negative bacteria could frequently lead to mortality. Bacterial endotoxins (lipopolysaccharides (LPS)) play a major role in pathogenesis of Gram-negative sepsis. The infection after injury, burn or surgery could lead to accumulation of released endotoxins in the bloodstream. LPS are able to interact with specific receptors on the surface of target host cells (monocytes/macrophages, neutrophils, endothelial cells, and so forth). After interaction with LPS, phagocytes change their morphological and functional properties, namely receptor expression, synthesis of proinflammatory cytokines and phagocytic activity. In the LPS signaling pathway, Toll-like receptor 4 (TLR4, CD284) is the main cell surface molecule inducing activation of NF-κB-dependent genes. Recent reports suggest that TLRs are involved in phagocytosis as the assistance receptors of phagocytes helping them to recognize invading bacteria or their molecular patterns [1,2]. Intensity of phagocytosis is potentiated by prior neutrophil exposure to endotoxins [3]. TLRs ligation by endotoxin molecules induces adaptor protein MyD88-dependent signaling involving IL-1 receptor-associated kinase (IRAK) and p38 mitogen-activated protein kinase leading finally to upregulation of phagocytosis-inducing genes. The integrated collaboration between TLR4 and phagocytosis has been shown early [4]. As shown during phagocytosis, TLR4 was only integrated in this functional property of the cells but not involved as the primary receptor [5]. It is well known that appropriate cell response to LPS is provided by engagement of CD14, TLR4 and CD11b receptors. Thus, we have studied the functional role of these receptors in phagocytosis of fluorescein-labeled *Escherichia coli *by human neutrophils in whole blood activated by S-form or Re-form endotoxins from *E. coli*.

## Methods

S-LPS from *E. coli *O55:B5 (Sigma) and Re-LPS from *E. coli *JM103 were obtained in our laboratory according to standard procedure. Fluorescein-labeled bioparticles *E. coli *K12 (Molecular Probes), mouse anti-human TLR4 mAbs HTA125 (IgG2a isotype; Serotec), anti-human CD14 clone UCHM-1 mAbs (IgG2a isotype; Sigma), and anti-human CD11b mAbs clone 44 (IgG1 isotype; Sigma). LPS were dissolved in pyrogen-free water to make 10 μg/ml stock solution, and sonicated vigorously for 20 minutes to increase LPS solubility. Experiments were performed in whole blood from nine donors (male, female, age 20 to 27). Blood was collected in sterile conditions into Monovette heparin-treated tubes 10 U/ml (Sarsted, Germany). Each tube contained 90 μl whole blood and 50 μl suspension of FITC-labeled bacteria (2 × 10^7 ^cells/ml; 1:10 leukocytes:bacteria ratio) was gently mixed and incubated for 30 minutes at 37°C. Then erythrocytes were lysed by hypotonic buffer for 5 minutes at room temperature. Samples were centrifuged (5 minutes, 200 × *g*, 21°C) and washed twice by cooled PBS containing 0.02% EDTA. The pellet was resuspended in 400 μl PBS-EDTA solution. To assess the influence of cell activation on phagocytosis the 100 ng/ml of S-LPS or Re-LPS were added before FITC-labeled bacteria and incubated during 30 minutes at 37°C. To determine the role of CD14, TLR4 and CD11b receptors in phagocytic activity, corresponding mAbs (1 μg, 30 minutes) were added to whole blood before cell activation by LPS and induction of phagocytosis. The phagocytic activity was monitored using the EPICS XL-MCL flow cytometer (Beckman Coulter). Results given as median fluorescence intensity. Phagocytic activity of cells without LPS activation or mAb treatment was established as 100%. Experiments were done two times using duplicate determinations in each experiment. The mean ± standard error was calculated using standard calculations available in the OriginPro spreadsheet. Statistically significant differences were determined by Student's *t *test.

## Results

**Figure 1:**
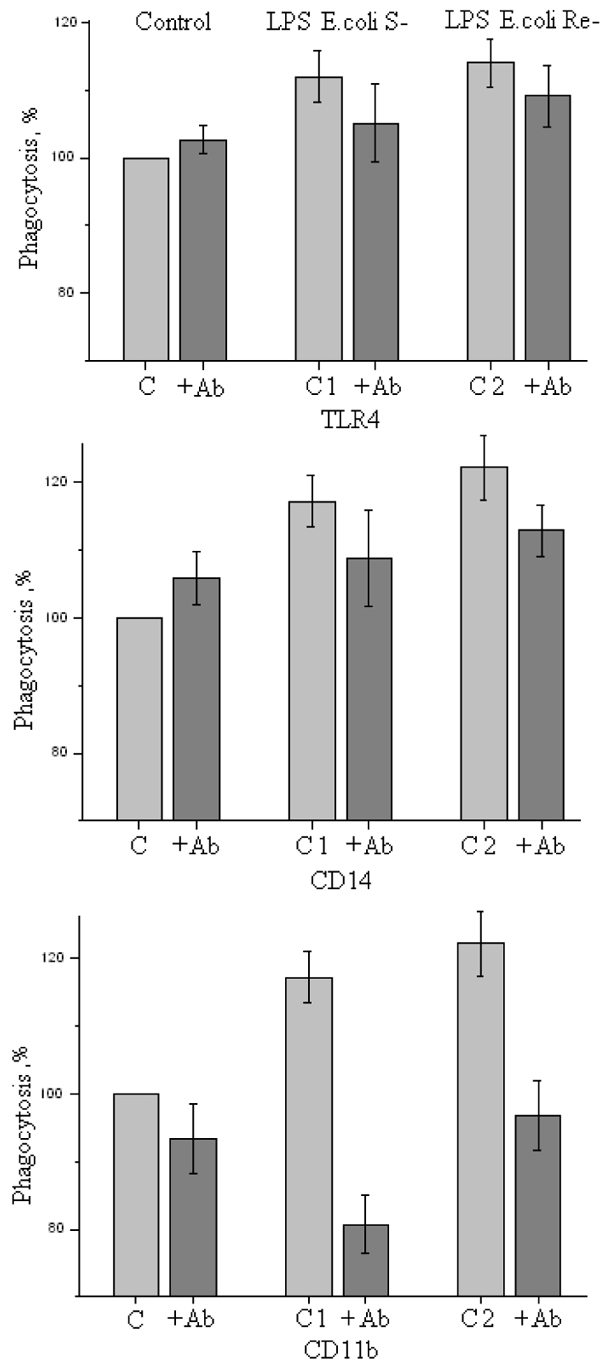
**Influence of antibodies against TLR4, CD14 and CD11b on neutrophil phagocytic activity in whole blood**. Control cells without any incentives (C), cells activated by S-LPS (C1) and cells activated by Re-LPS (C2).

Phagocytic activity of leukocytes was increased by pre-exposure of whole blood to S-LPS or Re-LPS (Figure 1). Pretreatment of whole blood by anti-CD14 or anti-TLR4 mAbs decreased to some extent the phagocytic activity of leukocytes activated by endotoxins independently of their glycoform (Figure 1). Significant inhibition of phagocytosis using anti-CD11b mAbs was achieved (Figure 1). Abs against CD11b more pronounced decreased S-LPS activated phagocytosis by 30% but were less effective in the case of Re-LPS (by 15%). It is known that CD11b receptors play an important role during phagocytosis of opsonized bacteria.

## Conclusion

LPS activate phagocytosis of Gram-negative bacteria independently of their glycoforms (Figure 1). The effect of HTA125 mAbs may be explained by their interaction with TLR4 leading to inhibition of LPS-induced signaling into the nucleus and blocking synthesis and surface expression additional receptors such as class A macrophage scavenger receptor (SR-A) [6] and CD11b [7] involved in phagocytosis. Activation of integrin-dependent signaling pathways can also be blocked by these HTA125 mAbs [8]. Anti-CD11b decreased most pronounced LPS-activated phagocytosis. Taking these results into consideration one can speculate that the Fc-chain of anti-CD11b (IgG_1_) mAbs through interaction with CD32A may mediate downregulation of TLR4 responses.
